# Clinical Observations on the Diseases of Children
*Journ. Hebdom. No. 91.


**Published:** 1830-10-01

**Authors:** 


### XLVIII.

### HOPITAL DES ENFANS.

### CLINICAL OBSERVATIONS ON THE DISEASES OF CHILDREN*

M. GUERSENT, from his official situation, has a wide field of observation open to him, in prosecuting the study of infantile diseases. Like his brethren on the French side of the channel, he has cultivated the department of morbid anatomy with a diligence which does him credit.

M. Guersent observes, that practitioners have considered the diseases of childhood in too exclusive a light. Growth and dentition are the scapegoats for all maladies, although children are not exempt from the same morbid processes that seize upon more advanced life, whilst they lay claim to others peculiar to themselves. Affections of the mucous membrane, and the class of ramollissemens are extremely common, and the morbid growths are not unfrequent. Phthisis pulmonalis, says M. G., is more frequent in infancy than at any other time of life; diseases of the circulatory system are rare, excepting, of course, malformations, and practitioners should be aware that the left ventricle is remarkably thick in early life, a circumstance which is often considered as an instance of hypertrophy; apoplexy is uncommon, but M. Guersent has seen a case of apoplexy of the spinal marrow, in a patient affected with diseased cervical vertebræ. He also maintains that many affections must be looked upon as nervous, inasmuch as no organic change is left behind. Children are subject to all kinds of neuralgia, and the genito-urinary system is liable to all the maladies which affect old age, excepting perhaps the chronic inflammation of the mucous membrane. Gangrene of the external parts of generation is frequent in young girls.

The diagnosis of infantile diseases presents many points of difficulty and doubt. The general sonorousness of the thorax, which even remains when the lungs are hepatized, may, if not understood, be productive of serious mistakes. Diseases steal on in the most insidious manner, pneumonia being ushered in with cephalic symptoms, and what appears to be a slight enteritis ending in the most alarming cerebral affection.

### MEMBRANOUS INFLAMMATION OF THE CHEEK.

This affection is particularly described by M. Guersent; it differs from the gangrena oris in being a much milder disease. It is more frequent in infancy than in adult age, on the right side of the mouth than the left. In the onset the mucous membrane of the cheek appears a little swollen, and beneath the epidermis there form little membranous patches, which run into one another, and spread over the cheek, the gums, the tongue, and the neighbouring parts. The glands of the neck are more or less enlarged, but as yet there is no ulceration of the cheek. In the second stage the cervical glands are more swollen, the face is tumid, the breath fœtid, the layers of false membrane become detracted and assume a greyish colour, and a copious sero-sanguineous discharge adds to the miseries of the little patient. There is usually little fever, the pain varies in different subjects, the salivary secretion is abundant. In the third stage the disease proceeds more slowly, and may terminate either in gangrene or in resolution. If the latter, the sloughs having come away the denuded surface is surrounded by a red-

* Journ. Hebdom. No. 91.

dish areola, which contracts the sore from the circumference to the centre, whilst absorption appears to be at work on the border of the membranous exudations. In other instances, when the affected surface is extensive, absorption proceeds in various places, even from the commencement of the malady. The disease may disappear and again return several times in the same individual. When the disease extends to the submucous cellular membrane it is liable to end in gangrene, but this never possesses the malignity nor advances with the rapidity of the true gangrena oris.

The disease is seldom attended with danger; is not, in M. Guersent's opinion, contagious; is chiefly developed in children debilitated by chronic affections, bad food, or hospital air; often follows the small-pox and other eruptive diseases; and appears to depend on a general alteration of the fluids. It seldom passes from the mouth to the pharynx, but one example of this kind was observed two years ago in the practice of M. Jadelot.

With respect to the treatment, topical applications only are important. If much pain is felt and the glands of the neck are considerably enlarged, the employment of leeches is advantageous. A mixture of muriatic acid and honey in equal parts, or made stronger if necessary, is, according to M. Guersent, the best application. He is likewise very partial to the nitrate of silver, taking care not to break it in the infant's mouth. If other diseases coexist with that under consideration, the general treatment must, of course, be adapted to the particular case.

---

